
JOURNAL INFORMATION
==============================
NLM Title Abbreviation: Wirtschaftsdienst
Journal ID: Wirtschaftsdienst
NLM Journal ID: 101086612

Wirtschaftsdienst (Hamburg, Germany : 1949)

ISSN: 0043-6275

ARTICLE INFORMATION
==============================
PMCID: PMC7896169
PMID: 33642642
DOI: 10.1007/s10273-021-2843-3
Article ID: 2843
Article version: 1
Subjects: Kurz Kommentiert

Digitale Infrastruktur verbessern!

Infrastrukturpolitik

Infrastructure Policy: Improving the Digital Infrastructure!

Kindsmüller Anna
Kaarst, Deutschland
Electronic publication date: 2021 Feb 20
Print publication date: 2021
Volume: 101
Issue: 2
First page: 76
Last page: 76
Copyright: © Der/die Autor:in(nen) 2021
License: Open Access: Dieser Artikel wird unter der Creative Commons Namensnennung 4.0 International Lizenz veröffentlicht (http://creativecommons.org/licenses/by/4.0/deed.de).
Open Access wird durch die ZBW–Leibniz-Informationszentrum Wirtschaft gefördert.
License URL: https://creativecommons.org/licenses/by/4.0/

issue-copyright-statement: © The Author(s) 2021

==============================

Die COVID-19-Pandemie hat durch Schließungen, das Zusammenbrechen von Lieferketten und Nachfrage zu erheblichen Verwerfungen in der deutschen Wirtschaft geführt und strukturelle Probleme deutlicher hervortreten lassen. Noch dreht sich die politische Debatte neben den wirtschaftlichen Auswirkungen des Coronavirus vor allem um mögliche Maßnahmen zur Pandemiebekämpfung. Solange das Virus präsent ist, hat die Eindämmung des Infektionsgeschehens Vorrang. Doch bald wird es um die Frage gehen müssen, welche wirtschaftlichen Lehren aus der Krise zu ziehen sind. Die dann zu treffenden politischen Entscheidungen werden die Wettbewerbsfähigkeit des Wirtschaftsstandorts Deutschland maßgeblich und langfristig beeinflussen. Hierbei wird es wichtig sein, wirtschaftspolitisch Prioritäten zu setzen, die schnellstmöglich die Weichen für eine zukunftsfähige Wirtschaft stellen, damit diese im internationalen Standortwettbewerb nicht abgehängt wird.

Über die deutsche Wettbewerbsfähigkeit wird schon seit einigen Jahren diskutiert. Neben hohen und unflexiblen Arbeitskosten führt auch das schlechte Abschneiden bei der digitalen Infrastruktur dazu, dass Deutschland als Wirtschaftsstandort in Rankings der internationalen Wettbewerbsfähigkeit zurückliegt (World Economic Forum, 2019). Mit Blick auf künftige politische Entscheidungen muss gefragt werden, ob und wie stark die COVID-19-Pandemie die Anforderungen deutscher Unternehmen an den Wirtschaftsstandort nochmals verändert. Solche Veränderungen in den Standortanforderungen der Unternehmen sind bisher zwar diskutiert, jedoch kaum an Daten gemessen worden, da die Daten hierfür einen direkten Vergleich von Standortanforderungen unmittelbar vor und nach dem Ausbruch der Pandemie ermöglichen müssen. Dies ist nur bei einigen wenigen Datensätzen der Fall. Die Umfragen zur Standortqualität bei Unternehmen einer Industrie- und Handelskammer (IHK Mittlerer Niederrhein, 2020), deren zwei Befragungswellen jeweils vor und nach dem Ausbruch/Lockdown im Frühjahr 2020 durchgeführt wurden, können genauere Einblicke in die durch COVID-19 veränderte Relevanz verschiedener Standortfaktoren bieten. Die Analyse zeigt, dass die Pandemie bei den Standortanforderungen deutscher Unternehmen eine Verschiebung ausgelöst hat (Kindsmüller, 2021): Standortfaktoren wie die Straßenanbindung, die in der Pandemie deutlich seltener genutzt wurden, verlieren signifikant an Bedeutung. So ist die Wahrscheinlichkeit für eine Bewertung dieses Faktors als sehr wichtig bei den befragten Unternehmer:innen – im Vergleich zu denen, die vor dem Ausbruch befragt wurden – um 52 Prozentpunkte geringer. Auch die Wahrscheinlichkeit für eine Bewertung des normalerweise wichtigen Themas Fachkräftemangel als sehr wichtig ist bei den nach dem Lockdown befragten Unternehmer:innen knapp 20 Prozentpunkte geringer. Ähnlich sieht es in einer Phase der zu großen Teilen stillstehenden Produktionen mit der Bedeutung der Energiepreise aus (minus 16 Prozentpunkte).

Die Analyse zeigt, dass während die genannten Wettbewerbsfaktoren durch die Pandemie an Bedeutung verlieren, die Anforderungen an die digitale Infrastruktur in Deutschland durch die Coronakrise weiter zunehmen. Sie wird bei den während der Pandemie befragten Unternehmen signifikant wichtiger eingestuft. Die Wahrscheinlichkeit für eine sehr hohe Relevanz steigt zusätzlich durch die Pandemie um 31 Prozentpunkte. Es kommt hinzu, dass auch die Zufriedenheit mit dieser Infrastruktur während der Pandemie gesunken ist.

Solche Zahlen sollten als dringender Appell gelesen werden, den Konsequenzen der Pandemie Rechnung zu tragen. Angesichts der Notwendigkeit und der gezeigten Dringlichkeit eines Ausbaus der digitalen Infrastruktur, der in Deutschland schon seit Jahren gefordert wird, eröffnet der in den Daten sichtbare Anstieg des Bedarfs vor allem eine Chance, den Ausbau der Strukturen jetzt schnellstmöglich voranzutreiben. Um diese Möglichkeit zu nutzen, muss der Ausbau nochmals verstärkt auf die politische Agenda gebracht werden. Dies gilt besonders auf kommunalpolitischer Ebene, auf welcher der Ausbau dieser Infrastruktur hauptsächlich umgesetzt wird. Auf dieser Ebene scheiterte der Ausbau bisher oft an der Bereitschaft der lokalen Unternehmen, die ihren Bedarf als zu gering wahrnahmen, um entsprechende Ausbaukosten mitzutragen. Die dargestellten Umfragedaten zeigen, dass hier eine Veränderung stattgefunden hat. Nun liegt die politische Chance darin, die aktuelle Bedarfssteigerung zu nutzen, um die eigenen Wirtschaftsstandorte wettbewerbsfähiger zu gestalten.

Anna Kindsmüller
Universität Münster
anna.kindsmueller@outlook.com


Literatur

IHK Mittlerer Niederrhein (2020), Daten der Standortumfragen Kempen und Meerbusch, erhoben durch die IHK Mittlerer Niederrhein, https://www.ihk-krefeld.de/7051 (20. Dezember 2020).
Kindsmüller, A. (2021), Die Pandemie verschiebt die Dringlichkeiten in der Infrastrukturpolitik, CIW Discussion Paper Series, 2.
World Economic Forum (2019), The Global Competitiveness Report 2019, http://www3.weforum.org/docs/WEF_TheGlobalCompetitivenessReport2019.pdf (28. Januar 2021).
Open Access wird durch die ZBW–Leibniz-Informationszentrum Wirtschaft gefördert.
